# Microfluidic generation of bacterial biohybrids for magnetic guidance and content release†

**DOI:** 10.1039/d5cc00449g

**Published:** 2025-07-14

**Authors:** Nina O’Toole, Matthew E. Allen, Claudia Contini, Yuval Elani

**Affiliations:** a Department of Chemical Engineering, Imperial College London South Kensington London SW7 2AZ UK y.elani@imperial.ac.uk; b Institute of Chemical Biology, Imperial College London, Molecular Sciences Research Hub London W12 0BZ UK; c Department of Chemistry, Imperial College London, Molecular Sciences Research Hub London W12 0BZ UK; d Department of Life Sciences, Imperial College London South Kensington London SW7 2AZ UK c.contini@imperial.ac.uk

## Abstract

Bacterial biohybrids use bacterial and synthetic components for biotechnological applications. Here, we outline an adaptable and high-throughput microfluidic platform to create microscale biocontained bacterial biohybrids enclosed in a hydrogel with magnetotactic and biosensing properties. The biohybrids are capable of magnetically driven motility, biochemical sensing and controlled cargo release. This approach enables the scalable fabrication of biocontained multifunctional biohybrids for potential industrial and biomedical applications.

## Abstract 2

We demonstrate a high throughput microfluidic method to produce bacterial biohybrids which are used for magnetotaxis, biosensing and cargo delivery.

## Graphical abstract

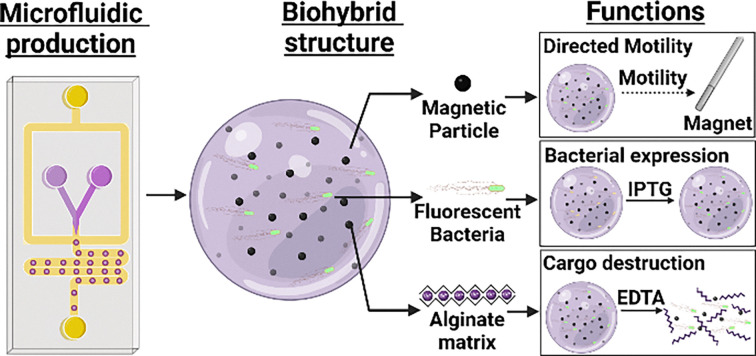

Bacterial biohybrids are micro-machines that combine bacterial components with synthetic and biological modules for biotechnological applications.^1,2^ Bacteria have many properties that are attractive for synthetic biology applications, including their engineerable self-regulating protein production systems,^3^ low maintenance culture techniques for rapid in-lab production,^4–6^ and chemotactic and motile machinery, which make them potential chassis for soft robotics.^1,4–8^ Such properties have led to their use as therapeutic and biosensing platforms,^1,6,9,10^ probiotics, immunostimulatory medicines, chemotherapeutic delivery vehicles^1,9–11^ and as targeted enzymatic reactors to treat infectious and metabolic diseases.^12^ Despite their advantages, bacterial biohybrids raise biosafety concerns, and specific containment requirements must be met, depending on the application.^13^ Regulations consider the risk of genetic exchange with pathogenic strains, antibiotic resistance and bacterial mutation.^12–14^ Edited strains can potentially alter ecosystems by competing with existing microbiota.^14^ In therapies there is a risk of bacterial escape to off-target bodily sites.^15^ Thus, there have been efforts to enhance biohybrid safety and targeting. A promising approach involves interfacing biohybrids with magnets to allow non-invasive control *via* forces applied externally to the body.^1^ This has been explored using aquatic bacteria, which are naturally magnetic due to ferritin packed organelles, as the biohybrid chassis,^5,7,10^ and by editing *E. coli* to produce these organelles.^16^ However, neither strategy resolves bacterial immunogenicity or the slow swimming speeds of aquatic bacteria at 37 °C.^1^

An alternate method uses hydrogels as the biohybrid chassis. These are selectively permeable matrices that can hold large cargo while maintaining dynamic aqueous environments.^17^ They can provide biocompatible structures that protect bacteria^18^ from the external environment and avoid immuno-activation.^19^ Furthermore, hydrogel particles can be produced in high throughput^20^ and incorporate biological and synthetic modules,^21^ making them an ideal chassis for biohybrid development.

Hydrogels have previously been interfaced with bacteria to improve their capabilities as microreactors and enhance chemotherapeutic biosafety.^12,19,22,23^ The advantages of hydrogels and magnetic targeting have also been combined to create sensing and motile biodevices.^24,25^

The increasing deployment of biohybrids, particularly those combining a hydrogel chassis with synthetic machinery, stresses the need for scalable and adaptable methods to construct new types of biohybrid. Ideally, such systems should integrate multiple functionalities—such as biosensing, motility, and on-demand cargo release—into a single, cohesive framework.

In this article, we present a high throughput and adaptable microfluidic approach for producing biohybrids that considers the above biohybrid safety and manufacturing aspects (Fig. 1a). The biohybrids comprise an alginate chassis containing nickel based microparticles and *E. coli* modified with a lac operon based GFP (green fluorescent protein)-expression circuit. These modules endow the biohybrids with chemically triggered biosensing, magnetotaxis, and controlled cargo release through hydrogel disassembly. We show that each component's function can be triggered individually or in tandem, highlighting the system's modularity. Furthermore, we demonstrate that the biohybrids can be magnetically guided to a target within a vasculature mimic where the chassis can be degraded to release the encapsulated cargo, illustrating this system's potential for therapeutic delivery and paving the way to develop increasingly complex and biosafe biohybrids.

**Fig. 1:**
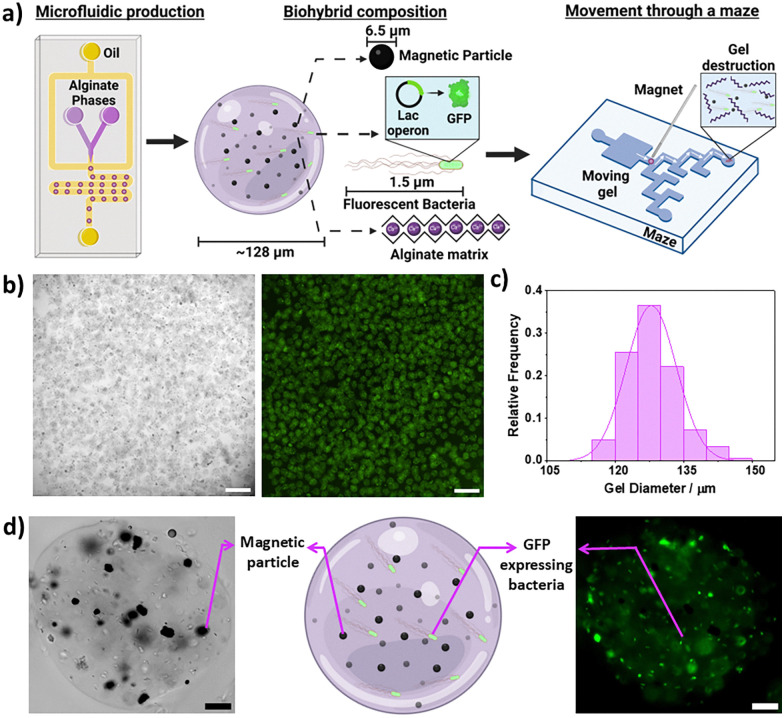
Summary of the biohybrid production process, composition, functional features and characterisation. (a) Biohybrids were synthesised by droplet microfluidics. Two alginate streams, both containing the biohybrids functional components, in this case magnetic particles and bacteria, converge at an oil flow focusing junction and were pinched into hydrogel precursor droplets. Gelation then occurred through ion exchange between the alginate phases. Biohybrids consisted of an alginate matrix containing magnetic particles and GFP-expressing bacteria. Biohybrids could be guided through a vasculature mimetic maze and chemically destroyed at a target location to release cargo. (b) Microscopy images of a population of biohybrids. Brightfield and fluorescence images are shown left and right respectively. Scale bars 500 μm. (c) A histogram showing the diameters of a population of biohybrids; the mean diameter was 128 μm with a standard deviation of 5.5 μm and a polydispersity index of 0.04. *N* = 181 biohybrid hydrogels were analysed. (d) Microscopy images with a diagram showing the biohybrids’ structure. A brightfield image is given on the left and a fluorescence image on the right. The magnetic particles and bacteria are visible in the images. Scale bars 20 μm.

The hydrogel biohybrids were prepared using a reported microfluidic method^20^ (Fig. 1a and Video S1, ESI†) on a PDMS chip (Fig. S1, ESI†). Gelation relied on an ion exchange reaction to crosslink the alginate matrix. This was selected over alternatives, such as acetic acid gelation, as it ensures mild gelation conditions that preserve cell viability.^20,26,27^ This method, CLEX (competitive ligand exchange crosslinking), uses two alginate phases, one containing Ca^2+^ chelated by EDTA and the other, Zi^2+^ chelated by EDDA. Once the phases mix, Zi^2+^ displaces Ca^2+^, due to EDTA's higher affinity for Zi^2+^, allowing Ca^2+^ to crosslink the alginate as the droplets pass through the chip.

Droplets were collected and resuspended in LB containing 100 mM CaCl_2_, the biohybrids were characterised. The biohybrid population (Fig. 1b and c) had a mean diameter of 128 μm with a standard deviation of 5.5 μm and PDI (polydispersity index) of 0.04. Particles with a PDI of <0.05 are regarded as monodisperse,^28^ emphasising this method's high throughput production of monodisperse biohybrids. Images of single biohybrids (Fig. 1d) show bacteria and magnetic particles successfully fixed in the gel while avoiding direct contact. Microscopy revealed that 14% (PDI 0.09) and 7% (PDI 0.08) of the hydrogel area was covered by cells and particles respectively (Fig S2, ESI†). By altering component concentrations during synthesis, the percentage encapsulation could be tuned (Fig. S3, ESI†). We observed that the biohybrids were not perfectly spherical and on occasion would merge during gelation. This may be due to droplets coming into contact, in the stabilization channels or during collection.

Once the biohybrids were synthesized, we demonstrated on-demand bacterial expression, activated independently to other biohybrid properties. On-demand expression is necessary to use biohybrids as biosensors or microreactors. Thus, biohybrids were equipped with *E. coli* that expresses GFP if exposed to the sugar IPTG (isopropyl β-d-1-thiogalactopyranoside)^29^ (Fig. 2a).

**Fig. 2:**
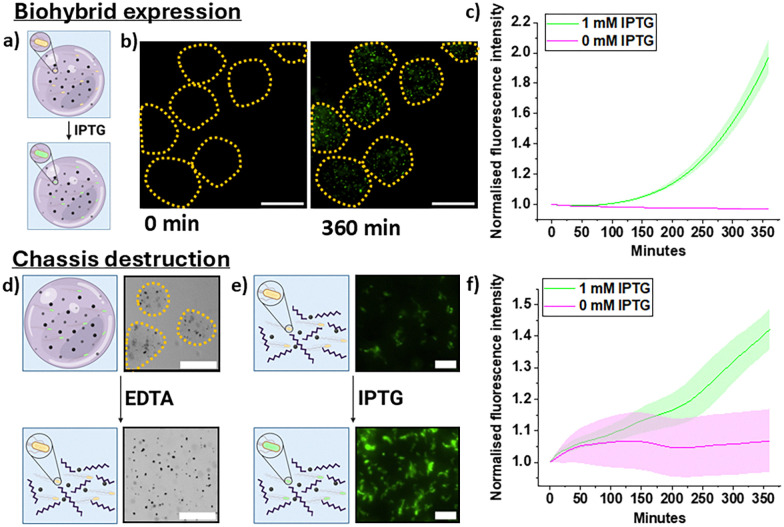
Bacterial expression and biohybrid dissolution. (a) A schematic depicting the effect of IPTG on biohybrids, bacteria express GFP following IPTG induction. (b) Microscopy images of biohybrids expressing GFP at 0 and 360 minutes after induction. Dotted lines show the positions of the hydrogel chassis. Scale bars 100 μm. (c) A graph of increasing fluorescence is seen over 360 minutes after IPTG addition. Error bars, represent one standard deviation of *N* = 50 gels at each timepoint. Error bars of the negative control are displayed but are imperceptible due to the low margin of error. (d) A schematic depicting the chassis destruction, accompanied by brightfield microscopy images of this process. Scale bars 100 μm. (e) A graphic depicting bacterial induction following chassis destruction, accompanied with fluorescent microscopy images of this at 0 and 360 minutes after IPTG induction. Scale bars 20 μm. (f) A graph showing that after hydrogel dissolution and IPTG induction, the fluorescence of the free bacteria increases. The change in fluorescence in the uninduced bacteria is attributed to leaky expression. Error bars represent the standard deviation from 5 regions containing free bacteria.

A fluorescence increase was seen within the biohybrids when exposed to 1 mM IPTG (Fig. 2b) (Video S2, ESI†). This fluorescence was localised to the bacteria, as GFP is a cytoplasmic protein. Alginate pores allow free diffusion of small molecules such as IPTG but prevent bacterial and particle escape.^27,30^ To ensure that IPTG fully permeated the gel matrix, diffusion of the similarly sized, fluorescent dye calcein was assessed, full diffusion was immediate (Fig. S4, ESI†). Once induced, fluorescence increased exponentially as biohybrids were monitored (Fig. 2c). The standard deviation of expression increased over time, indicating that GFP expression varies between biohybrids as is to be expected of different microbial populations. Biohybrids not treated with IPTG showed negligible fluorescence increase, with a final normalized fold change of ∼0.002 compared to the initial value. This was similar to the expression in the bulk (Fig. S5, ESI†).

To test whether GFP expression was affected by the magnetic particles, gels were produced containing bacteria without the particles (Fig. S6, ESI†). The trend in expression was unaltered for these gels, confirming that particles did not impact expression. This series of experiments verified bacterial viability within the biohybrid and showed controlled expression when encapsulated. We also investigated biohybrid stability, a crucial property for potential therapeutic applications.^31^ Over 12 hours, colony growth was seen, reaffirming viability (Fig. S7 and S8, ESI†) (Video S3, ESI†) whilst the gel remained intact. Small fibrils can be seen forming at the gel edges, indicating that a small number of peripheral cells may escape.

This matched the results seen in other studies^27,32^ and confirmed that the alginate gel was an excellent chassis for the biohybrids. To illustrate the biohybrid potential as a bacterial delivery vehicle, we assessed expression after gel disassembly. This strategy is relevant to therapeutic applications where bacteria need direct access to a target.^33^ To destroy the chassis, 110 mM EDTA was added to the biohybrids in solution. EDTA chelates the Ca^2+^ linking the alginate, destroying the matrix (Fig. 2d).

Upon EDTA addition, the biohybrids lose their spherical shape and, the functional modules are dispersed within the media. After disassembly, we added 1 mM IPTG to the free bacteria to ensure that they retained their expressive capacity (Fig. 2e and f) (Video S4, ESI†). Cells were also incubated in bulk with EDTA to test if this affected expression (Fig S9, ESI†). In both instances, the cells retained viability and expressive capacities hours after EDTA exposure. Like encapsulated cells, free bacteria expressed GFP upon IPTG addition and the limited expression seen in uninduced bacteria was attributed to leaky expression.^34^

After displaying the biohybrids’ expressive capacity, we worked towards demonstrating that they could be magnetically manipulated. The incorporation of magnetic particles into the biohybrid design allows them to be moved towards a target under the control of non-invasive magnetic fields, increasing their therapeutic relevance.^35,36^ Initially we confirmed the biohybrids’ magnetically driven motility by observing their motion towards a 1 Tesla magnetic rod (Fig. S10, ESI†) (Video S5, ESI†). The biohybrids were placed in a maze with channel widths and heights of 1 mm, mimicking the small veins of the body^37,38^(Fig. S11, ESI†). Biohybrids were then directed to a destination well with the same magnet (Fig. 3a) (Videos S6 and S7, ESI†). The biohybrids only moved in response to the magnet and moved towards the directed channels with a high degree of specificity. The magnet was held 2–5 mm diagonally above and away from the channels and gels, to guide them through the maze. At times, the gels would stall at the uneven channel sides, requiring magnetic reorientation to pass through the channel. At the destination well, we demonstrated EDTA disassembly (Fig. 3b) (Video S8, ESI†). The chassis was destroyed in seconds freeing the cargo within the destination well, as seen by the components spreading in solution, emphasising the potential for targeted cargo delivery.

**Fig. 3:**
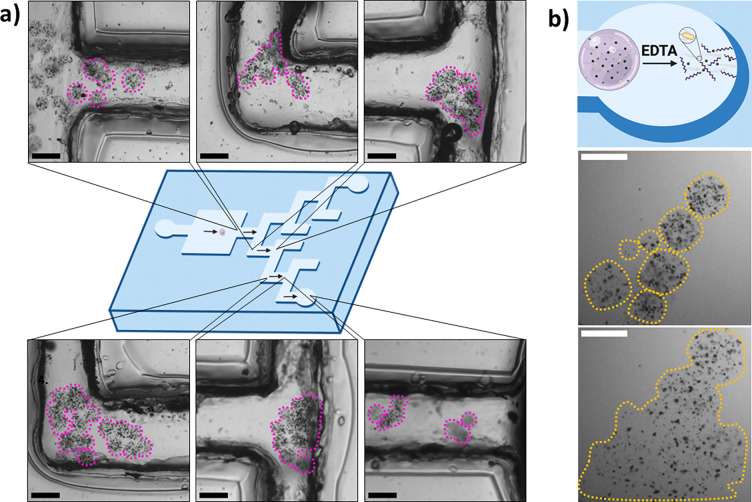
Biohybrid magnetotaxis. (a) Graphic alongside images of the biohybrids passing through a maze, controlled by a magnet. Brightfield images show a group of biohybrids, outlined in purple, at different positions in the maze. Scale bars 200 μm. (b) Biohybrid destruction at the end of the maze on exposure to EDTA. The top panel shows a graphic of destruction. Middle and bottom panels show images of intact and destroyed biohybrids, outlined in yellow, before and after EDTA addition respectively. Scale bars 200 μm.

In summary, we have shown a high throughput adaptable microfluidic method to produce biohybrids containing modified bacteria that respond to biochemical cues, and microparticles that confer magnetic control. We showed that the components could be activated by different triggers orthogonally, a quality necessary to develop more complex biohybrid microsystems.

Due to the production method, additional modules such as responsive lipid vesicles, for secondary cargo release,^21^ or alternate bacterial strains^39^ could also be incorporated. This could increase biohybrids’ functionality and allow for the use of more complex bacterial control systems, such as toggle switches, to improve control over therapeutic release.^40^

Our production method allows the biohybrids’ size and shape to be altered by adjusting the microfluidic design.^41^ This could potentially adjust the speed and efficiency of biohybrid movement through fluids,^42^ impacting drug delivery efficiency.

We also showed that the biohybrids could be magnetically steered through a vasculature mimic to a target, where cargo could be released or synthesized. On occasion, the method led to droplet merging. While this was thought to have a minimal effect on movement, it could be mitigated by collecting gels after a longer period of gelation. Encapsulating the bacteria in a biocompatible chassis, protected them from activating the immune system and destruction, increasing the probability of cell survival in the body and lowering the risks of sepsis and patient toxicity.^1,43,44^ Coating biohybrids in a biocompatible material or limiting their delivery times can be used to avoid peripheral cell escape. Additionally, a single biohybrid can transport a cell population to a target site and deliver a bulk therapeutic dose, rather than relying on many free swimming cells to reach targets to deliver equivalent payloads.^5,7,9,45^ By using a chassis that can be degraded on demand, bacteria can be positioned directly or indirectly positioned at target sites, increasing this system's applications for drug delivery.^46^ EDTA does not hinder expression, but with prolonged exposure, expression plateaus faster, thus, washing away EDTA is suggested for optimal performance. To circumvent this an enzyme such as alginate lyase could be used for degradation.

To increase the therapeutic potential of the biohybrids, their stability under biological conditions could be assessed further: the guidance system could be optimised for clinically relevant magnetic systems^5^ and additional modules could be added.^47^ We anticipate that this proof-of-concept biohybrid platform will help to generate increasingly complex soft matter biohybrid systems that will aid the advancement of biohybrids as therapeutic biosensors and delivery systems.

## Conflicts of interest

There are no conflicts to declare.

## Supplementary Material

CC-061-D5CC00449G-s001

CC-061-D5CC00449G-s002

CC-061-D5CC00449G-s003

CC-061-D5CC00449G-s004

CC-061-D5CC00449G-s005

CC-061-D5CC00449G-s006

CC-061-D5CC00449G-s007

CC-061-D5CC00449G-s008

CC-061-D5CC00449G-s009

## Data Availability

The data supporting this article have been included as part of the ESI.†
