# Supplementary material for: Microfluidic generation of bacterial biohybrids for magnetic guidance and content release
Source: Chem Commun (Camb). 2025 Jul 14;61(65):12155–8. doi: 10.1039/d5cc00449g (PMC12257921; doi:10.1039/d5cc00449g)
Supplement: CC-061-D5CC00449G-s001 [file CC-061-D5CC00449G-s001.pdf]

Supplementary Information (SI) for Chemical Communications.
This journal is © The Royal Society of Chemistry 2025

Supplementary Information

Microfluidic generation of bacterial biohybrids for magnetic guidance and content release

Author list

Nina O'Toole [1], Matthew E. Allen [1,2,3], Claudia Contini[4*], Yuval Elani[1,2*]

[1]Department of Chemical Engineering, Imperial College London, South Kensington, London SW7 2AZ
[2]Institute of Chemical Biology, Imperial College London, Molecular Sciences Research Hub, London, W12 0BZ
[3]Department of Chemistry, Imperial College London, Molecular Sciences Research Hub, London, W12 0BZ
[4]Department of Life Sciences, Imperial College London, South Kensington, London SW7 2AZ

*Email: c.contini@imperial.ac.uk, y.elani@imperial.ac.uk

Materials and Methods

Materials

MagneHis™ Ni Particles were purchased from Promega. QuickPick™ 1-M Magnetic tool was purchased from BN Business. 2 mm continuous cast acrylic Polymethyl methacrylate (PMMA) was obtained from Clarex 001, Weatherall Equipment and Instruments Ltd, UK. 3M™ High Performance Acrylic Adhesive 200MP was sourced from 3M, USA. SYLGARD™ 184 silicone elastomer base and SYLGARD™ 184 silicone elastomer curing agent were obtained from The DOW chemical company, Europe. Ethylenediaminediacetic acid (EDDA) was obtained from Tokyo chemical industry co., LTD. Calcium chloride was sourced from VWR international BVBA. Rainex was bought from Kraco Car Care International Ltd. 176 x 52 x 1 mm Microscope slides were bought from Marienfeld. pET His6 GFP TEV LIC cloning vector for GFP expression was obtained from Addgene (Plasmid #29663). BL21-DE3 *E. coli* were acquired from New England Biolabs. All other chemicals including Sodium Alginate, Zinc Acetate, 4- (2-hydroxyethyl)-1-piperazineethanesulfonic acid (HEPES), Ethylenediaminetetraacetic acid (EDTA), Mineral oil, Span 80, Kanamycin, Ampicillin, Isopropyl ß-D-1-thiogalactopyranoside (IPTG), Luria-Bertani (LB), calcein and LB agar were obtained from Sigma Aldrich chemicals (Gillingham, UK).

Alginate solution preparation

Two alginate phases were prepared, with one composed of 2% alginate, 84 mM EDTA, 84 mM Calcium chloride and 40 mM HEPES at pH 6.4 and the other made up of 2% alginate, 84 mM EDDA, 84 mM Zinc Acetate and 40 mM HEPES at pH 6.4. MagneHis Particles were added to each solution bringing the final solution concentrations to 5 mg/ml magnetic particles.

Cell culture

The bacterial strain used in this study was BL21-DE3 *E. coli* transformed with pET His6 GFP TEV LIC cloning vector for GFP expression. Plated colonies were grown on LB Agar containing 50 µg/ml Kanamycin. Liquid cultures of GFP expressing BL21-DE3 *E. coli* were prepared from a plated colony and grown overnight in LB broth doped with 50 µg/ml Kanamycin incubated at 37 °C at 200 RPM. Cells from the liquid culture were split and grown until they reached OD 0.38. Cultures were then aliquoted and incubated at 4 °C. Aliquots were centrifuged for 10 min at 4000 RCF and resuspended in Zinc-EDDA and Calcium-EDTA alginate phases bringing the final OD in each alginate phase to 0.76.

Microfluidic chip production

Chip designs were inspired by previous work[1,2]. Designs were used to produce a photomask and cast to a silicon wafer using soft lithography[1,3]. This involved depositing a photoresist (SU-8 3050, Kayaku Advanced Materials, MA, USA) of 100 µm depth using a spin coater onto a silicon wafer (Inserto). The silicon wafer was then baked before exposure to a UV light (365 nm, 300 mJ cm^{-2}) through an acetate photomask (Micro Lithography services, UK) containing the microfluidic device designs. A second post exposure bake was then performed before the unexposed features were eliminated using propylene glycol monomethyl ether acetate developer and an Isopropyl alcohol rinse. Finally, the patterned wafers were silanised with trichloro(1H, 1H, 2H, 2H-perfluorooctyl)silane under an overnight vacuum.

The cleaned patterned wafers had degassed Polydimethylsiloxane (PDMS) Sylgard 184 elastomer (10:1 Elastomer: Curing agent) poured onto them before the wafers were cured for 3 hours at 60 °C. The PDMS containing the device pattern was removed from the wafer before 1.5 mm holes were punched into the PDMS for inlets and outlets. The PDMS containing the microfluidic device design was then bonded to a glass slide through exposing both the glass slide and patterned side of the PDMS to plasma (Harrick Plasma, NY, USA) for 90 seconds before contacting the surfaces together. This sealed the microfluidic channels. Before use the microfluidic devices were left overnight to ensure complete bonding between the PDMS and glass slide.

Biohybrid production

Calcium – EDTA and Zinc – EDDA alginate phases containing MagneHis particles and bacteria at a concentration of ~ 3×10^8 cells/ml, along with an oil phase containing mineral oil and 5% v/v Span 80, were passed into appropriate inlets of the microfluidic device (**Fig. S1**) through Polyethylene tubing (Kinesis, UK). A pressure pump (Elveflow, Paris, France) was used to control the flow rates of the alginate and oil phases. The alginate phase flow rates were altered to ensure an equal flow of both phases while the oil phase flow rate was larger than the alginate phase flow rates to ensure droplet formation occurred. The produced hydrogel biohybrids were collected in an Eppendorf, centrifuged at 4000 RCF for 10 minutes to remove oil and resuspended in LB containing 100 mM Calcium Chloride. The $CaCl_2$ was added to prevent any biohybrid disassembly from occurring.

For all experiments save one control, concentrations of ~ 3×10^8 cells/ml and 5 mg/ml were used for Bacteria and MagneHis particles respectively. For controls where the magnetic particles and bacteria were reduced, 1 mg/ml of MagneHis particles and a 5-fold dilution of bacteria were used in the alginate phases.

Maze production

The maze design was composed of multiple T-junctions and based on previous work by Salek et al[4]. Designs were drawn in CoralDraw graphic suite. Chips were cut using a VLS 2.30, Universal Laser Systems, Austria, laser cutter on 2 mm hard continuous PMMA cast acrylic with double sided acrylic adhesive attached to one side of it. Adhesive was used to attach the maze to a glass slide (**Fig. S7**). Chips were checked for leaks before use and treated with the hydrophobic glass cleaner, Rainex, to prevent gel adherence to the glass bottom.

Timelapse microscopy, image processing and data analysis

Microscopy images were taken on a Nikon Eclipse Ti2-U inverted microscope with a CoolLED pE-300^{white} and a Nikon DS-Qi2 camera with a variety of objectives (4X, 10X, 20X) under brightfield illumination and the GFP filter for fluorescence.

Images were analysed using FIJI and graphs were plotted in Origin or using python packages (matplotlib and seaborn). Statistical analysis was performed with the SciPy.stats library in python where an unpaired *t*-test with unequal sample variances was used. The *p* values were set to the following significance levels: * < 0.05, ** < 0.01, and *** < 0.001.

To determine the percentage area covered by magnetic particles or bacteria, hydrogel area was measured, and the proportion of this area covered by magnetic particles or the fluorescent signal from the bacteria, was used to calculate the % area in relation to hydrogel size.

To obtain normalized fluorescence datasets for biohybrids, spheres were drawn around a selection of from 13 populations taken over 2 days. Pixel fluorescence of spheres was measured every 10 minutes for 360 minutes. Pixel fluorescence for every gel and background measurement was normalized to:

$$F = \frac{i}{i_0}$$

(Equation 1)

F is the normalized fluorescence, i is the raw fluorescence value at time t and i_0 is the raw fluorescence value at time t=0. Final graphs were obtained by plotting the average and standard deviation of all these averaged values at each time point. For the negative control the data was processed in the same way on uninduced gels for 6 populations.

To obtain the normalized fluorescence datasets for individual bacteria linear ROI's were drawn over individual bacterial cells and adjacent background regions for 6 populations. Data was normalized and averaged as described previously.

The diameter of gels was determined from the area of spheres drawn around 181 gels.

The fluorescence change in hydrogels without magnetic particles present was normalised using equation 2. F is the normalised fluorescence, i is the raw fluorescence value at time t, i_0 is the raw fluorescence value at time t=0, $i_{background}$ is the fluorescence background value at time t and $i_{0\,background}$ is the background fluorescence value at time t=0.

$$F = \frac{i - i_{background}}{i_0 - i_{0\,background}}$$

(Equation 2)

Bulk bacteria characterisation

Bulk bacteria at OD 0.38 were suspended in LB containing 100 mM $CaCl_2$ and 110 mM EDTA. 1 mM IPTG was additionally added to positive samples to induce GFP expression. Bacteria solutions were then placed in a 96 wellplate and incubated at 37°C on a CLARIOstar plate reader and shaken between measurements. Samples were monitored for 360 minutes with measurements of GFP fluorescence (excitation 470 nm, emission 515 nm) taken every 20 minutes. Samples were normalised to the initial fluorescence intensity using equation 1.

Magnetic motion experiments

For recording the brightfield magnetic movement videos and imaging magnetically induced motion, a Nikon TE2000-U inverted microscope with a Ximea MQ013MG-E2 camera and 4x objective or a Nikon Eclipse Ti2-U inverted microscope with a CoolLED pE-300^{white}, a Nikon DS-Qi2 camera and 6x magnification were used.

Magnetic motion experiments in bulk were performed by placing a 1 Tesla magnetic rod approximately 1 mm away from the centre of the sample well. The motion of the biohybrids was recorded before the manual tracking tool on ImageJ was used to select the biohybrid centres at 0.5 s intervals to obtain the X and Y coordinates at each timestep. The coordinates were used to calculate the total distance the biohybrids travelled using the following equation (equation 3). In the equation, X and Y are the x and y coordinates of the analysed biohybrid, n is the number of intervals evaluated and i is the current timestep.

$$Total\ distance = \sum_{i=1}^{n} \sqrt{(X_{i+1} - X_i)^2 + (Y_{i+1} - Y_i)^2}$$

(Equation 3)

The motion experiments in the maze were performed by filling the maze with LB containing 100 mM Calcium Chloride. The biohybrids were then placed in the large square well at the beginning of the maze. To guide the biohybrids towards a targeted destination well, a 1 Tesla magnetic rod was placed

a few mm away from the biohybrids. Videos were recorded whilst the biohybrids were under the action of the magnet.

Supporting figures

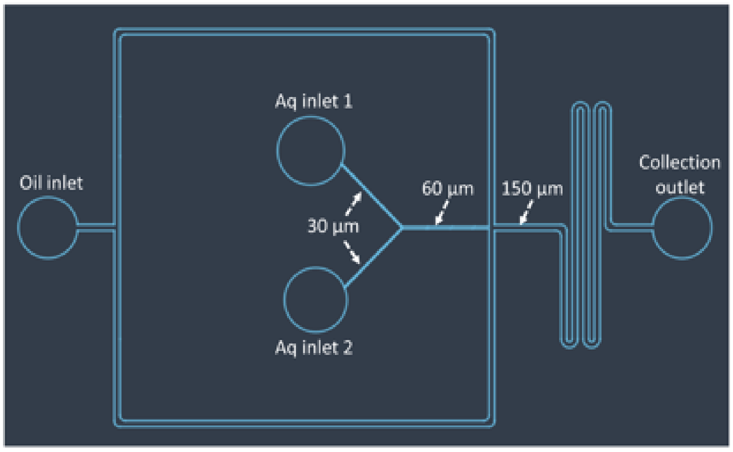

Figure S1: Schematic demonstrating dimensions of the microfluidic chip used to create biohybrids. The chip consists of two aqueous inlets through which Zinc-EDDA and Calcium-EDTA alginate solutions can be fed. Droplets are formed at the flow focussing junction where the aqueous inlets meet the oil phase. Following this the droplets gelate and the produced biohybrids are collected at the outlet. The channel depth was always 100 µm.

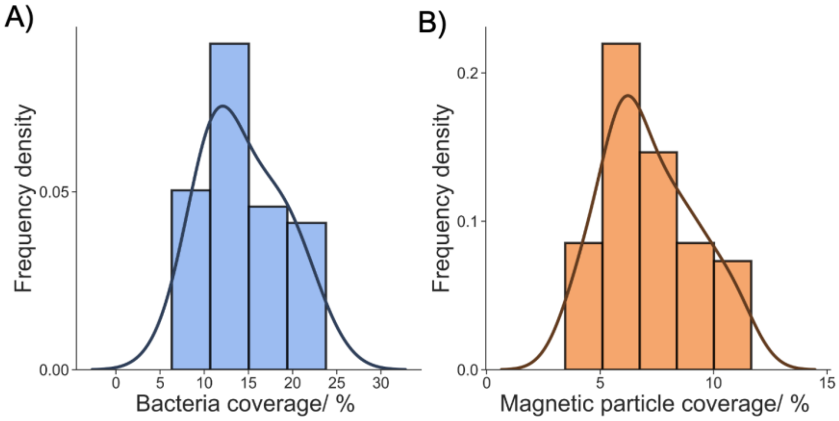

Figure S2: Histograms of bacteria and magnetic particle encapsulation in the biohybrids. A) A histogram of bacteria coverage in a population of biohybrids after 1 hour of incubation with 1 mM IPTG. The average coverage was 14% and the polydispersity index was 0.09. **B)** A histogram of magnetic particle coverage in a population of biohybrids. The average coverage was 7% and the polydispersity index was 0.08. N=50 bacterial biohybrids were analysed for each histogram. The polydispersity index values demonstrate a degree of heterogenous encapsulation particles throughout the bacterial biohybrid as the polydispersity indexes are slightly higher than the 0.05 polydispersity threshold for monodispersity.

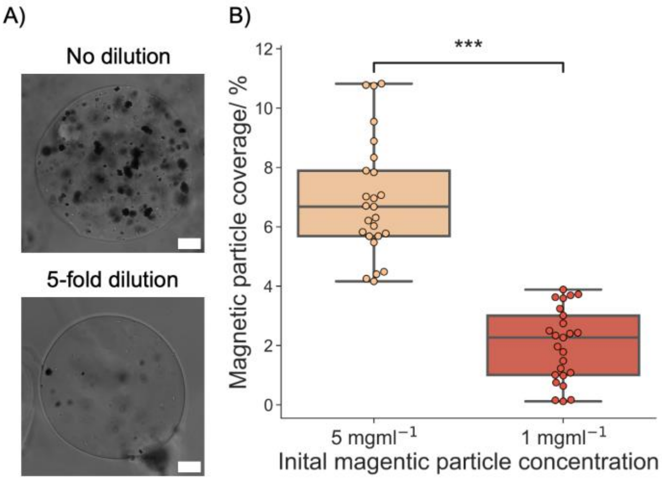

Figure S3: Varying magnetic particle and bacteria loading in the biohybrids A) Brightfield images of bacterial biohybrids produced with the initial magnetic particle/ bacteria concentrations and a 5-fold dilution of the magnetic particle/ bacteria concentrations. Upon dilution significantly less magnetic particles and bacteria are present within the hydrogel chassis. The scale bars are 20 μm. **B)** Bar charts comparing the magnetic particle coverage in the biohybrids. N=25 bacterial biohybrids were analysed for each plot. The p value was 5.23×10^{-13}. This shows that different populations of biohybrids can be produced by varying the concentrations of bacteria and magnetic particles added to the alginate solutions.

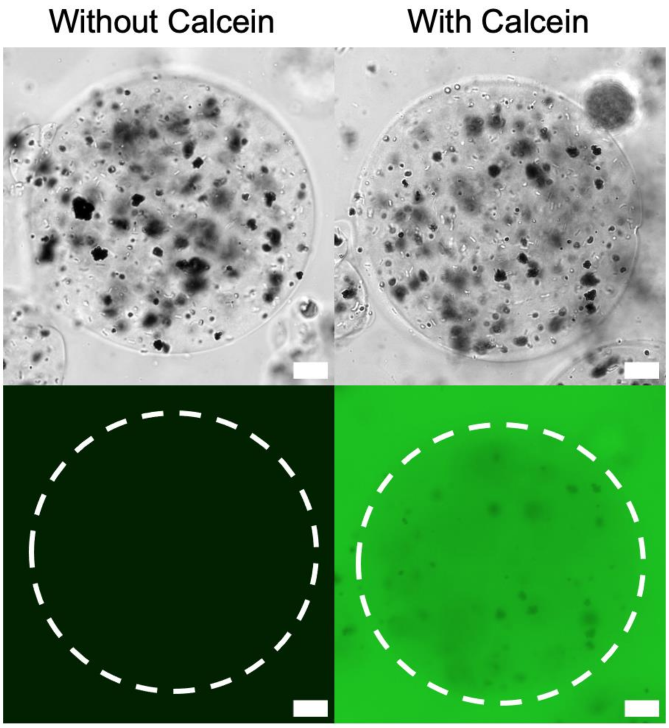

Figure S4: Calcein permeation in bacterial biohybrids. Brightfield and fluorescence microscopy images showing bacterial biohybrids before and after 0.25 mM Calcein addition. On the additional of the small molecule Calcein (MW 622.55 Da) a fluorescent signal can be seen throughout the bacterial biohybrid immediately, indicating the free permeation of Calcein through the hydrogel chassis. The dotted circles in the fluorescence images indicate the position of bacterial biohybrids. The scale bars are all 20 μm.

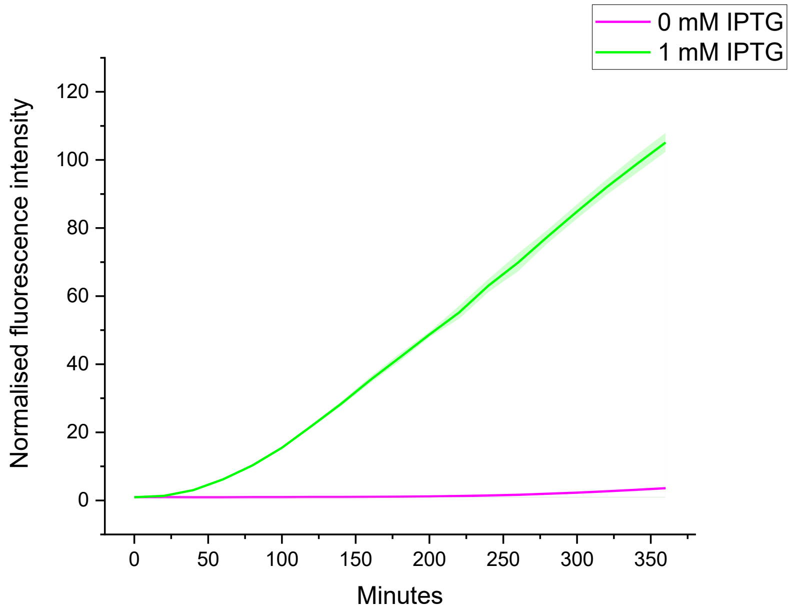

Figure S5: Expression of *E. coli* cells in bulk. Populations of *E. coli* were treated with 1 mM of IPTG or an equivalent volume of water. GFP expression was monitored over a period of 6 hours in a CLARIOstar where upon IPTG addition an increase in fluorescence was observed. Without IPTG minimal fluorescence was seen accounting for leaky GFP expression. This demonstrates that GFP expression can be induced in bulk under the control of IPTG. Error bars represent the standard deviation of n=4 wells containing bacteria per condition.

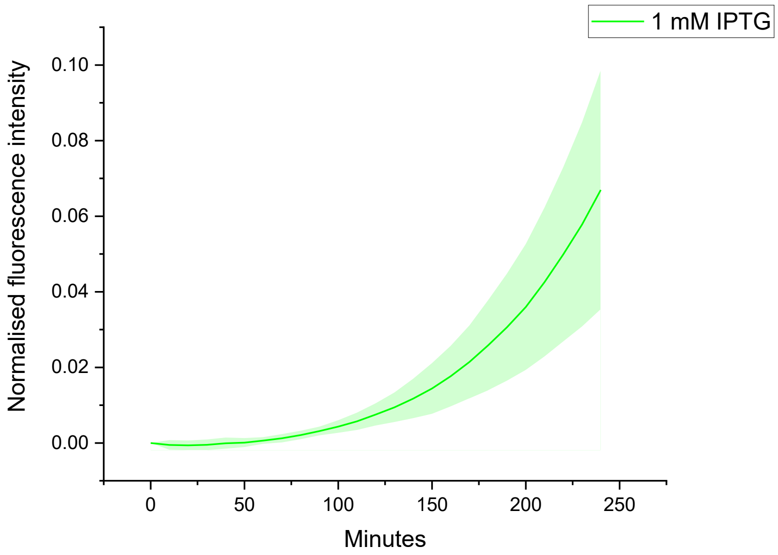

Figure S6: Graph depicting the normalised fold change in fluorescence of biohybrids that do not contain magnetic nanoparticles. The increase in fluorescence is similar to the biohybrids containing magnetic particles, indicating that the magnetic particles do not impact on the bacterial expression. The error bars are the standard deviation of n=108 biohybrids

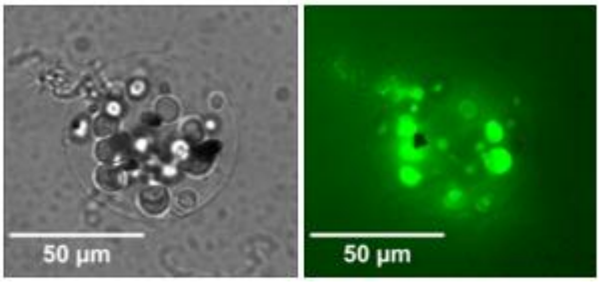

Figure S7: Growth of bacteria within the biohybrids. Brightfield and fluorescence images of biohybrids taken after 18 hours incubated with LB, 100 mM $CaCl_2$ and 1 mM IPTG. The bacteria had grown into larger structures visible in both imaging channels, further demonstrating bacteria activity and stable of encapsulation of the bacteria within the hydrogel chassis. The scale bars are 50 µm.

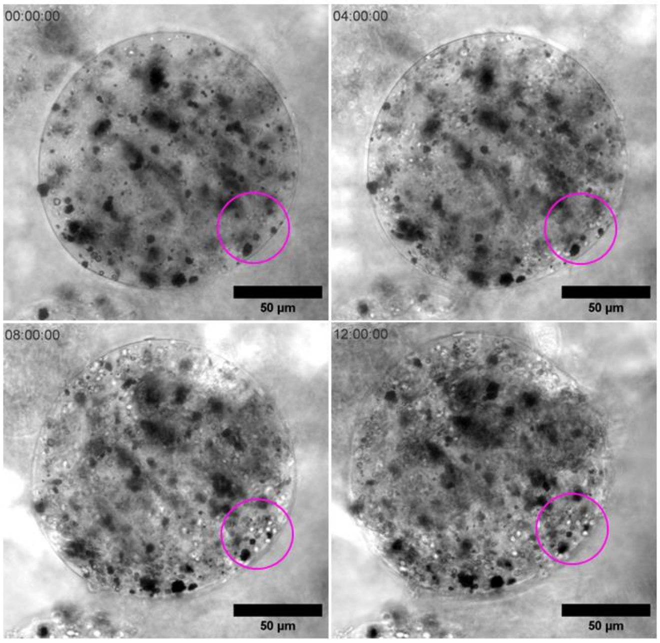

Figure S8: Growth of bacteria within the biohybrids. Brightfield images of biohybrids taken over a 12-hour period incubated with LB, 100 mM $CaCl_2$. Images show the same gel at 0, 4, 8 and 12 hours. The bacteria grow into larger colonies over time but remain encapsulated within the gel, showing that the biohybrid is mechanically stable over this timeframe despite bacteria proliferation. The growth of bacterial colonies in a specific gel region is highlighted with a purple circle. The scale bars are 50 µm.

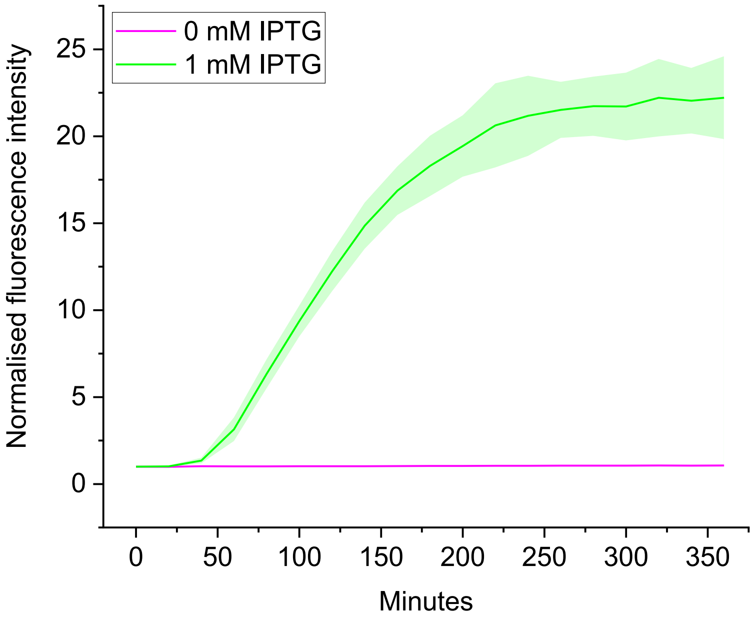

Figure S9: Expression of *E. coli* cells incubated with EDTA in bulk. Populations of *E. coli* were incubated in LB+ 100 mM CaCL2. Samples were treated with 110 mM EDTA and 1 mM of IPTG or an equivalent volume of water. GFP expression was monitored over a period of 6 hours in a CLARIOstar. Upon IPTG addition an increase in fluorescence was observed until ~150 minutes where expression tapers off leading to a plateau. Without IPTG minimal fluorescence was seen. This demonstrates *E. coli* retain their ability to express GFP in bulk after treatment with EDTA. Error bars represent the standard deviation of n=5 wells containing bacteria per condition.

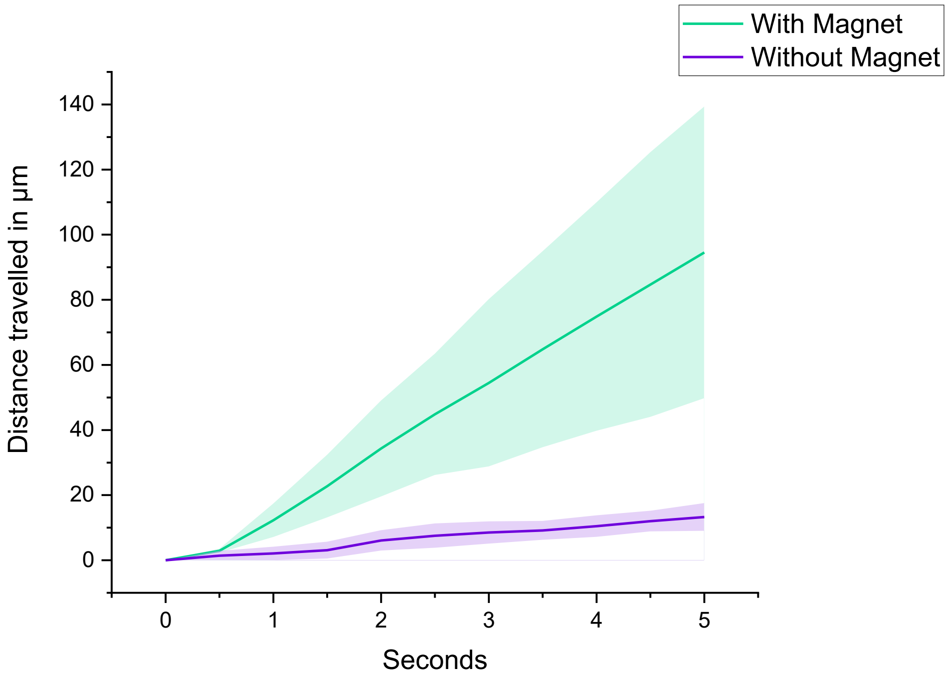

Figure S10: Magnetically induced motion of the biohybrids. Plot showing the movement of the biohybrids with and without application of the magnetic field. On magnetic field application, movement of the biohybrids is observed, showing that the biohybrids move in response to a magnetic field. The error bars on the graph are the standard deviation of the movement of n=5 biohybrids per condition.

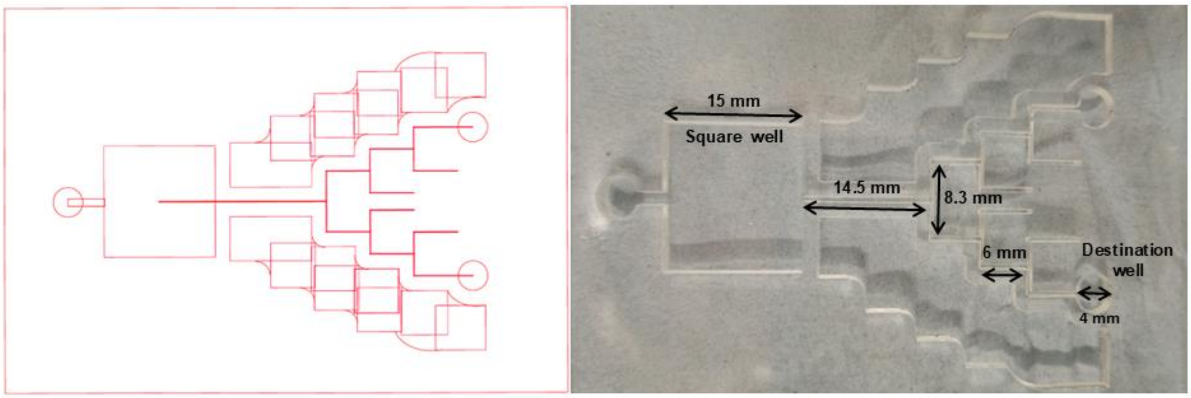

Figure S11: Image of the magnetic maze used for guidance experiments. The maze consists of a square well to deposit gels in bulk and a series of diverging channels through which biohybrids can be guided leading up to 2 destination wells. The left image displays the CAD schematic used to laser cut the maze. Right shows an image of the maze taken with a phone camera. The channel dimensions and start/ destination wells are labelled.

Supplementary videos

Video S1: Production of biohybrids in the microfluidic device. Zinc-EDDA and Calcium-EDTA alginate solutions, containing magnetic particles and bacteria, are fed from two different central alginate inlets. Alginate solutions are mixed in a straight channel before being pinched off into droplets by an oil phase at a flow focusing junction. The video pans to show droplets passage through a stabilization channel where the CLEX ion exchange reaction and gelation can occur. Biohybrids are then flown through a final collection outlet. Scale bar in bottom right represents 500 μm.

Video S2: Expression of biohybrids containing GFP-producing *E. coli* over a period of 240 minutes. At the beginning of the video, outlines of gels and magnetic particles can be seen. As time passes the bacterial cells begin to express GFP which can be seen as pockets of fluorescent material appearing in the gels. Scale bar represents 100 μm.

Video S3: Growth of bacterial colonies within a biohybrid over 12 hours. The video taken in brightfield reveals bacterial colonies growing in size while anchored within the gel chassis. At about 9 hours small fibrils can be seen forming at the edge of the gel area. Scale bar represents 50 μm.

Video S4: Expression of GFP-producing *E. coli* over a period of 240 minutes after their release from biohybrid carriers with IPTG. Biohybrids were treated with 110mM EDTA to trigger dissolution of the alginate gel carrier through calcium chelation. The video displays fluorescence microscopy images of free *E. coli* cells that were treated with IPTG following carrier dissolution. Cells were excited with a GFP filter and imaged every 10 minutes for a period of 240 minutes.

Video S5: Movement of biohybrids with a magnet. Biohybrids were placed in a microscopy well and a magnet was placed ~0.1 cm from the well. The video displays the biohybrids movement towards the magnet for 5 seconds alongside tracks showing their progression over time. The scale bar is 100 μm.

Video S6: Movement of Biohybrids through the maze entrance. The video displays the entrance channel of the PMMA maze where a collection of biohybrids can be seen to be moving into the maze. The panning of the video into the channel shows that the biohybrids have moved into the maze. The scale bar is 250 μm.

Video S7: Movement of Biohybrids through a maze junction. The video displays a junction within the PMMA maze where a collection of biohybrids can be seen to be moving selectively towards the bottom channel of the maze. The downwards panning of the video shows that the biohybrids have moved into the lower channel at the junction. The scale bar is 250 μm.

Video S8: Biohybrid dissolution with EDTA at end of maze. 110 mM of EDTA was added to biohybrids in the destination well in the maze. The hydrogel chassis dissolved releasing the magnetic particles and the bacteria. Scale bar represents 200 μm.

Acknowledgements

A special thanks to Gabriela Sachet Fernández for the provision of the bacterial strains. Additional thanks to Karen Keyue Zhu for assistance with CLARIOstar experiments. This work was supported by a UKRI Future Leaders Fellowship, grant reference number MR/S031537/1 (awarded to Y.E.); an Engineering and Physical Sciences Research Council (EPSRC) Centre for Doctoral Training Studentship from the Institute of Chemical Biology (Imperial College London) (awarded to M.E.A.), and a BBSRC Discovery Fellowship, grant reference number BB/W009323/1 (awarded to C.C.). For the purpose of open access, the author(s) has applied a Creative Commons Attribution (CC BY) license to any Author Accepted Manuscript version arising. Graphics were created in BioRender. Elani, Y. (2024) https://BioRender.com/y07a410.

Author contributions

N.O designed and performed experiments, analyzed the data and wrote the manuscript, M.E.A designed experiments, helped analyze the data and revise the manuscript, C.C and Y.E designed experiments and helped revise the manuscript.

References

1 M. E. Allen, J. W. Hindley, N. O'Toole, H. S. Cooke, C. Contini, R. V. Law, O. Ces and Y. Elani, Proc. Natl. Acad. Sci., 2023, 120, e2307772120.

2 A. G. Håti, D. C. Bassett, J. M. Ribe, P. Sikorski, D. A. Weitz and B. T. Stokke, Lab. Chip, 2016, 16, 3718–3727.

3 T. Trantidou, M. Friddin, Y. Elani, N. J. Brooks, R. V. Law, J. M. Seddon and O. Ces, ACS Nano, 2017, 11, 6549–6565.

4 M. M. Salek, F. Carrara, V. Fernandez, J. S. Guasto and R. Stocker, Nat. Commun., 2019, 10, 1877.
